# Acute myocardial infarction prognosis prediction with reliable and interpretable artificial intelligence system

**DOI:** 10.1093/jamia/ocae114

**Published:** 2024-05-28

**Authors:** Minwook Kim, Donggil Kang, Min Sun Kim, Jeong Cheon Choe, Sun-Hack Lee, Jin Hee Ahn, Jun-Hyok Oh, Jung Hyun Choi, Han Cheol Lee, Kwang Soo Cha, Kyungtae Jang, WooR I Bong, Giltae Song, Hyewon Lee

**Affiliations:** School of Computer Science and Engineering, Pusan National University, Busan 46421, Republic of Korea; School of Computer Science and Engineering, Pusan National University, Busan 46421, Republic of Korea; Department of Cardiology, Medical Research Institute, Pusan National University Hospital, Busan 49241, Republic of Korea; Department of Cardiology, Medical Research Institute, Pusan National University Hospital, Busan 49241, Republic of Korea; Department of Cardiology, Medical Research Institute, Pusan National University Hospital, Busan 49241, Republic of Korea; Department of Cardiology, Medical Research Institute, Pusan National University Hospital, Busan 49241, Republic of Korea; Department of Cardiology, Medical Research Institute, Pusan National University Hospital, Busan 49241, Republic of Korea; College of Medicine, Pusan National University, Gyeongsangnam-do 50612, Republic of Korea; Department of Cardiology, Medical Research Institute, Pusan National University Hospital, Busan 49241, Republic of Korea; College of Medicine, Pusan National University, Gyeongsangnam-do 50612, Republic of Korea; Department of Cardiology, Medical Research Institute, Pusan National University Hospital, Busan 49241, Republic of Korea; College of Medicine, Pusan National University, Gyeongsangnam-do 50612, Republic of Korea; Department of Cardiology, Medical Research Institute, Pusan National University Hospital, Busan 49241, Republic of Korea; College of Medicine, Pusan National University, Gyeongsangnam-do 50612, Republic of Korea; Gupo Sungshim Hospital, Busan 46581, Republic of Korea; Division of Cardiology, Department of Medicine, Busan Veterans Hospital, Busan 46996, Republic of Korea; School of Computer Science and Engineering, Pusan National University, Busan 46421, Republic of Korea; Center for Artificial Intelligence Research, Pusan National University, Busan 46421, Republic of Korea; Department of Cardiology, Medical Research Institute, Pusan National University Hospital, Busan 49241, Republic of Korea; College of Medicine, Pusan National University, Gyeongsangnam-do 50612, Republic of Korea

**Keywords:** acute myocardial infarction, explainable artificial intelligence (XAI), interpretable machine learning, counterfactual explanation, electronic health records (EHR)

## Abstract

**Objective:**

Predicting mortality after acute myocardial infarction (AMI) is crucial for timely prescription and treatment of AMI patients, but there are no appropriate AI systems for clinicians. Our primary goal is to develop a reliable and interpretable AI system and provide some valuable insights regarding short, and long-term mortality.

**Materials and methods:**

We propose the RIAS framework, an end-to-end framework that is designed with reliability and interpretability at its core and automatically optimizes the given model. Using RIAS, clinicians get accurate and reliable predictions which can be used as likelihood, with global and local explanations, and “what if” scenarios to achieve desired outcomes as well.

**Results:**

We apply RIAS to AMI prognosis prediction data which comes from the Korean Acute Myocardial Infarction Registry. We compared FT-Transformer with XGBoost and MLP and found that FT-Transformer has superiority in sensitivity and comparable performance in AUROC and F1 score to XGBoost. Furthermore, RIAS reveals the significance of statin-based medications, beta-blockers, and age on mortality regardless of time period. Lastly, we showcase reliable and interpretable results of RIAS with local explanations and counterfactual examples for several realistic scenarios.

**Discussion:**

RIAS addresses the “black-box” issue in AI by providing both global and local explanations based on SHAP values and reliable predictions, interpretable as actual likelihoods. The system’s “what if” counterfactual explanations enable clinicians to simulate patient-specific scenarios under various conditions, enhancing its practical utility.

**Conclusion:**

The proposed framework provides reliable and interpretable predictions along with counterfactual examples.

## Introduction

Acute myocardial infarction (AMI) is a critical cardiac event with a high mortality risk and complications, requiring immediate treatment, including revascularization of the infarct-related artery. Although advances in modern percutaneous coronary intervention (PCI) and medical therapies over the past decades have led to a continuous decrease in mortality, the risk still remains relatively high.^1^ Furthermore, the development of heart failure (HF) after AMI significantly impacts patient prognosis, tripling overall mortality, and quadrupling cardiovascular mortality.^2^ Therefore, contemporary studies have underscored the need for timely intervention and tailored interventions to mitigate AMI mortality and HF progression.

To deliver timely intervention and personalized therapy for AMI patients, it is crucial to assess prognosis and predict short-term mortality. Clinical experts often have employed scoring systems like the thrombolysis in myocardial infarction score^3^^,^^4^ and the Global Registry of Acute Coronary Events (GRACE) score^5^^,^^6^ to achieve this. They utilize basic admission, peri-procedural characteristics, and laboratory findings to predict in-hospital mortality^3^^,^^4^ and relatively short-term mortality^6^ as well as long-term mortality with revised GRACE score version 2.0.^7^ These systems were utilized in the past to aid in the initial triage of ACS (Acute coronary syndrome) patients, supported by moderate evidence in previous guidelines.^8^ However, they do not encompass a wide spectrum of patients, including those who may experience in-hospital complications beyond the PCI event. Furthermore, since they were developed in the early 2000s, their applicability is compromised by their limited accuracy (Refer to the previous works such as^9^^,^^10^ and the empirical results in Appendix Table S1). These limitations restrict their utility to long-term prognosis prediction and individualization of care.^11^

Recent studies have proposed advanced machine learning (ML) techniques using electronic health record (EHR) data to assess risk and predict mortality in AMI cases.^9^^,^^10^^,^^12^^,^^13^ Despite their promising results, there are some critical limitations in using their results for clinical decision-making. First, most ML approaches are black-boxed. This characteristic causes significant challenges for the experts to understand, validate, and justify the predictions and outcomes of the ML models.^14–16^ Additionally, these approaches often provide only predictions without tangible insights for clinical action and their predictions do not reflect the true likelihood despite their common usage as such. For example, while an artificial intelligence (AI) system may predict a patient’s mortality as 90%, the actual likelihood can vary significantly from the prediction, making it challenging for experts to rely on these methods.

We believe that to be successful in clinical decision-making with the AI system, the individual strengths of the clinician and the AI should come together to optimize the joint decision outcome.^17^ To this end, the AI system should maintain the transparency of its decision by providing insightful explanations to allow clinicians to appropriately apply their knowledge to improve the final decision. In addition, the prediction should represent the true correctness likelihood so that clinicians can trust the AI system’s prediction. In this regard, we propose the following criteria for a reliable and interpretable AI system (RIAS) for clinical decision-making. (1) The system should explain both the mechanisms and the reasons for the outcomes it produces.^18–22^ (2) The system should offer additional insights to the users on how users can achieve desired outcomes alongside predictions.^19^^,^^20^ (3) Their predictions should reflect the actual likelihood for classification tasks.^18^^,^^19^^,^^22^

Our study introduces the RIAS—an end-to-end framework that is designed with the above principles at its core. RIAS generates an optimized model for a given dataset and ensures both reliability and interpretability in outcomes. With RIAS, we analyze AMI prognosis data from the Korean Acute Myocardial Infarction Registry (KAMIR) and provide some valuable insights. Concretely, we apply RIAS to in-hospital, 6-month, and 12-month mortality prediction tasks for the AMI patients. RIAS is available at https://github.com/Alcoholrithm/RIAS.

## Methods

### Description of the data

The study utilized data from the Korean Acute Myocardial Infarction Registry (KAMIR) for the years 2015-2018.^23^ KAMIR is a nationwide registry on AMI with 53 participating centers with facilities for PCI and on-site cardiac surgery. A trained coordinator collected data using a standardized case report form and protocol. This protocol conformed to the ethical guidelines of the 1975 Declaration of Helsinki, as reflected by prior approval from the human research committee of each participating institution. The Institution Review Board of Pusan National University Hospital approved this study (IRB number: 2202-004-111). Clinical outcomes are defined as clinical events including in-hospital, 6-month, and 12-month mortality.

#### Characteristics of the cohort

The cohort’s baseline characteristics were as follows. The mortality rates for each clinical event are 3%, 5%, and 6% for in-hospital, 6-month, and 12-month mortality, respectively. The mean age was 64.3 years (±12.5 years). 76.9% were male. The prevalent past medical conditions included hypertension (50.1%), current smoking (36.7%), diabetes mellitus (27.9%), and dyslipidemia (14.4%). Nearly half of the cohort had ST-segment elevation AMI (STEMI) (47.1%). The majority (81.5%) presented with Killip class I, indicating a favorable clinical status. Initial thrombolysis therapy was administered to 1.5% of STEMI patients, while the overall rate of PCI was 91.1% (97.0% in the STEMI subgroup and 86.3% in the non-STEMI subgroup). Among complications during hospitalizations, the following were frequent as sequences: cardiogenic shock (5.1%), hemodynamically significant ventricular tachycardia/ventricular fibrillation (3.9%), new-onset heart failure (2.3%), ≥ BARC 2 bleeding (2.1%), and acute kidney injury (0.9%). Other variables include laboratory findings during hospitalization, echocardiography results, angiographic, and procedural characteristics, as well as discharge medications. In this patient cohort, ACE inhibitors or ARBs were prescribed for over 72% of patients, 74% of patients were prescribed beta-blockers, and nearly all patients (98% for aspirin, 97% for P2Y12 inhibitors) were taking aspirin and P2Y12 inhibitors (clopidogrel, ticagrelor, and prasugrel), and over 90% of patients were prescribed a statin. This indicates that the patients in this cohort serve as excellent examples of adherence to guideline-directed therapy. A summary of these baseline characteristics is provided in Table 1.

**Table 1.** Characteristics of the cohort.

Feature	Mean or proportion	Feature	Mean or proportion
Age	64.28 ± 12.51	Systolic blood pressure (mmHg)	131.08 ± 29.53
Female (%)	23.1 (3615)	Diastolic blood pressure (mmHg)	78.75 ± 18.24
Killip Class at admission (%)		Heart rate (bpm)	79.38 ± 19.48
I	81.50 (12 737)	Current smoker (%)	36.68 (5732)
II	8.27 (1293)	Angiographic and procedural findings	
III	5.46 (854)	Puncture route	
IV	4.77 (745)	Femoral	39.77 (6215)
Height (cm)	165.73 ± 28.26	Radial	50.02 (7817)
Weight (kg)	66.89 ± 15.14	Number of involved vessels (%)	
Past history (%)		1	3.24 (507)
Hypertension	49.91 (7800)	2	44.79 (7000)
Diabetes mellitus	27.94 (4367)	≥ 3	46.91 (7331)
Dyslipidemia	14.42 (2253)	Target vessel (%)	
Myocardial infarction	6.96 (1088)	LM	2.54 (397)
Angina	7.92 (1238)	LAD	42.71 (6674)
Heart failure	1.33 (208)	LCX	16.07 (2511)
Cerebrovascular accident	6.42 (1003)	RCA	29.82 (4660)
Atrial fibrillation on initial ECG (%)	2.5 (391)	Treatment of target vessel (%)	90.87 (14,201)
Initial ECG presentation		Result of PCI (%)	
STEMI	47.16 (7370)	Successful	89.87 (14 045)
NSTEMI	52.84 (8258)	Suboptimal	0.83 (130)
Final diagnosis		Failed	0.34 (53)
STEMI	46.46 (7260)	Index procedure (%)	90.9 (14,206)
NSTEMI	51.34 (8024)	Status of revascularization (%)	
UAP	0.58 (90)	Complete	57.74 (9023)
Laboratory findings at presentation	13.85 ± 2.09	Partial	27.57 (4308)
Hb (g/dL)		Use of IVUS (%) 22.74	(3554)
Creatinine (mg/dL)	1.27 ± 1.14	Use of FFR (%)	1.7 (265)
Peak Troponin I (ng/mL)	42.2 ± 59.51	Use of OCT (%)	3.16 (494)
Peak CK-MB (ng/mL)	107.2 ± 131.36	Complications during hospitalization (%)	12.48 (1951)
hsCRP (mg/dL)	13.9 ± 2.09	Discharge medication (%)	
LDL-cholesterol (mg/dL)	109.71 ± 37.81	Aspirin	98.16 (15 341)
HDL-cholesterol (mg/dL)	43.19 ± 10.35	Beta-blocker	73.71 (11 520)
Triglyceride (mg/dL)	142.48 ± 94.67	Clopidogrel	56.64 (8851)
Echocardiography result	51.62 ± 10.35	Ticagrelor	50.5 (7892)
Left ventricular ejection fraction (%)		Prasugrel	6.9 (1078)
Left ventricular end systolic volume (mL)	47.99 ± 23.37	ACEi inhibitors	33.17 (5184)
Left ventricular end diastolic volume (mL)	96.70 ± 30.26	ARB	39.68 (6201)
Mitral regurgitation grade (%)		Statin	92.17 (14 405)
I∼II	41.62	CCB	10.16 (1588)
≥III	1.91	Anticoagulant	5.05 (789)

Statistics are mean±SD (standard deviation) for continuous features, and proportions (counts) for categorical features.

Abbreviations: ECG, electrocardiogram; MI, myocardial infarction; STEMI, ST segment elevation MI; NSTEMI, non-ST segment elevation MI; UAP, unstable angina pectoris; CK, creatine kinase; MB, myocardial band; hsCRP, high-sensitivity C-reactive protein; LDL, low-density lipoprotein; HDL, high-density lipoprotein; LM, left main; LAD, left anterior descending; LCX, left circumflex; RCA, right coronary artery; PCI, percutaneous coronary intervention; IVUS, intravascular ultrasound; FFR, fractional flow reserve; OCT, optical coherence tomography; ACEi, angiotensin-converting enzyme; ARB, angiotensin II receptor blocker; CCB, calcium channel blocker.

### RIAS framework

**Figure 1.**
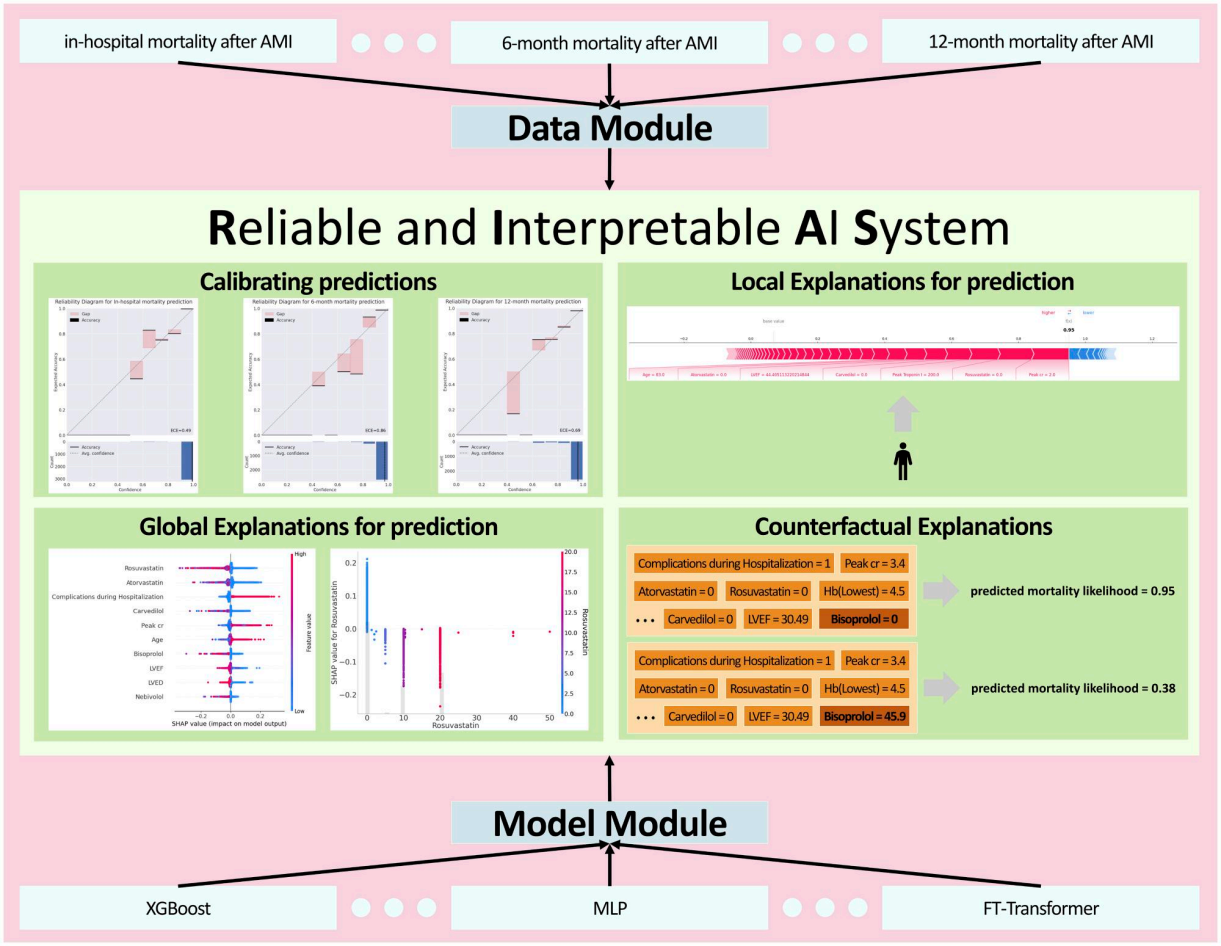
Overview of reliable and interpretable artificial intelligence system framework.

To achieve a RIAS for medical experts, we developed the RIAS framework, which consists of the following components. Note that, RIAS can be applied to any model or dataset as it is a model-agnostic and dataset-agnostic framework, which allows for wider application. Figure 1 demonstrates the overview of RIAS.

#### Automatic optimizing step

The hyperparameter of the model is one of the most crucial factors to achieve an accurate model. Therefore, we combined an automatic hyperparameter optimizing step with RIAS. RIAS finds out the best hyperparameters using Optuna^24^ during predefined trials for given search ranges and given an evaluation metric. In this study, we used F1 Score for Optuna^24^ and the search ranges for each model are shown in the supplementary Appendix.

#### Global and local explanations using SHAP values

A prior comprehensive work^25^ identifies that the explanation of explainable AI (XAI) must be intuitively interpretable and easily understandable. Furthermore, it should provide global and local views because features in EHR data have different semantic meanings depending on the view. Therefore, we chose to use SHAP to provide global and local explanations of RIAS, as it meets the requirements outlined.^26^^,^^27^ While there are other XAI methods available, such as data dimensionality reduction, knowledge distillation, and rule extraction, we found SHAP to be the most suitable for our needs. SHAP employs game theory approaches to explain the outputs of any ML model.^26^ It assigns each feature an importance value, called SHAP values, for a given prediction. The SHAP values denote the contributions of each feature to the prediction. They allow us to obtain a comprehensive understanding of the prediction of the model from both local and global perspectives. The global explanations of RIAS are derived by analyzing the SHAP value over the training data, and the local explanations accompany the prediction made by RIAS. RIAS uses PermutationSHAP to compute the SHAP value in a model-agnostic way for broader applications.

#### Deeper insights for desired outcomes

“What if” to change the patients to the preferred outcome is the major interest of clinicians. To achieve this, RIAS provides counterfactual explanations, which are hypothetical examples that offer guidance to clinicians on how to improve the predicted patient outcomes. The counterfactual explanations of RIAS inform not only which features need to change, but also “how much should be changed.” For medical professionals, counterfactual explanations should provide rich examples in a variety of scenarios and must be feasible. In the comprehensive benchmark,^28^ DiCE^29^ shows its competitive strengths, especially in the diversity of counterfactual explanations, in this criterion among various counterfactual explanation methods. In addition, DiCE^29^ can handle categorical features, such as sex, that are critical to EHR data, while some methods cannot.^30^ In this regard, RIAS uses DiCE^29^ to generate counterfactuals. Note that RIAS always generates feasible counterfactual examples by adding user constraints for the generation. To generate counterfactual explanations, the user must select which features to perturb that clinicians can influence such as treatment or medication, and set constraints for them, such as the dosage of a drug.

#### Reliable prediction

**Figure 2.**
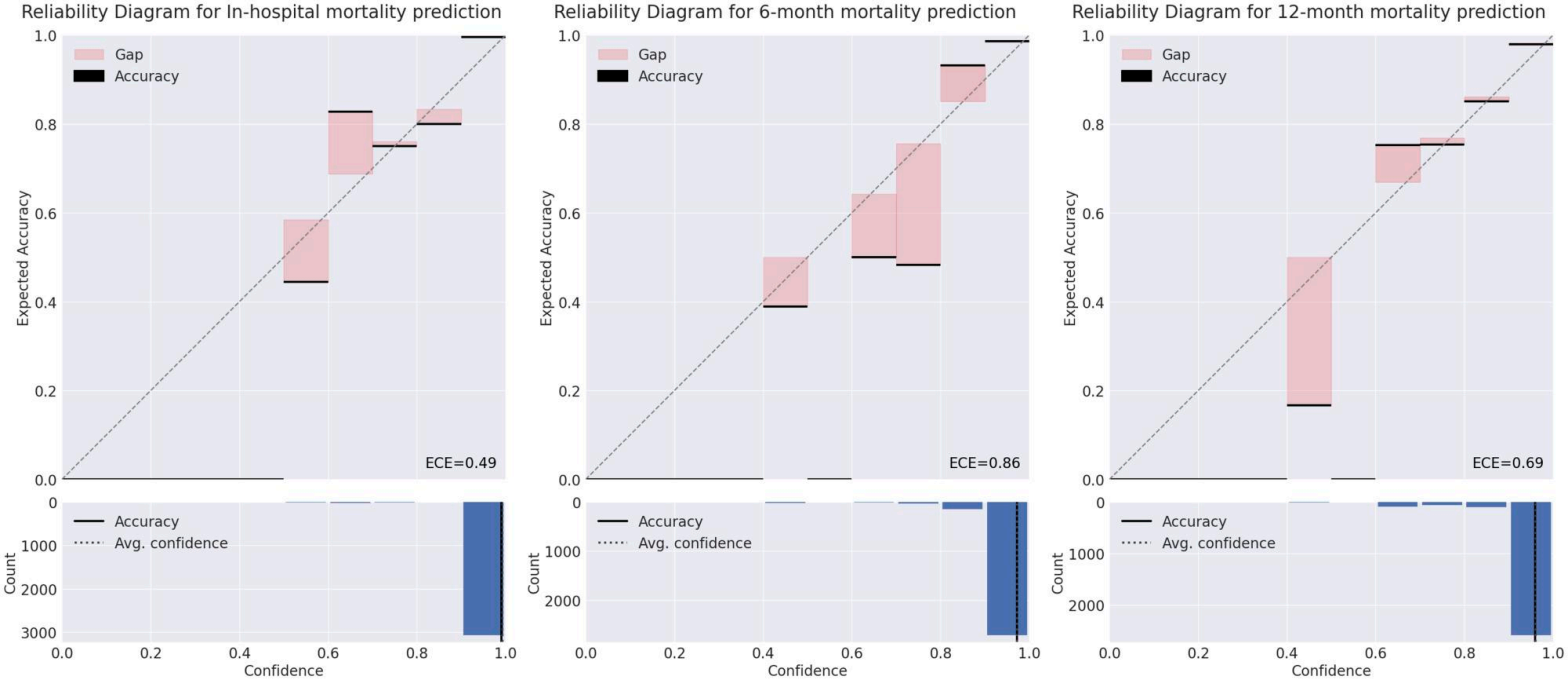
Reliability diagram for each task. The diagram illustrates how well the predicted likelihoods (confidences) of a model correspond to the actual outcomes. In the diagram, predictions are grouped into bins based on their predicted probability. If the model is perfectly mirroring the actual likelihood then the diagram should plot the identity function. Any deviation from a perfect diagonal (red bars) represents discrepancy. These discrepancies are measured using expected calibration error (ECE, the details regarding ECE are in supplementary Appendix). The closer to zero, the better ECE.

In practical applications where the AI system derives decisions, the decisions must not only be accurate but also should indicate potential inaccuracies. For those scenarios, the AI system typically outputs a value between 0 and 1 for each class. A value of 1 indicates that the system is certain that the sample belongs to that class, while a value of 0 indicates the opposite. These output values are often interpreted as the confidence of the system and users regard it as likelihood. However, such confidence values are mostly unreliable.^31^ Therefore, RIAS calibrates the confidence to more accurately represent the true correctness likelihood, enhancing the system’s trustworthiness for clinicians (Figure 2). Consequently, practitioners can determine how much they need to believe the prediction of the AI system. The comparison before and after applying the confidence calibration is shown in the supplementary Appendix.

#### Experimental setups of RIAS in this study

Unless otherwise noted, we set the following setups to show the empirical results using RIAS in this article. We reserved 20% of data as test data to evaluate the AI system and the rest is used for training and analyzing features. We adopted 5-fold cross-validation to tune the model using RIAS and tuned each model with 100 trials using Optuna.^24^ Due to the class imbalance, all splits are done in a stratified fashion. Since the dataset is highly imbalanced, we used scale pose weight for XGBoost and WeightedRandomSampler for neural networks. Additionally, the dataset was split in a stratified manner, considering the class imbalance. We used histogram binning for confidence calibration after the AI model generated its prediction. For reproducibility, we fix the random seed as 0.

## Results

### Performance comparison between ML models

Recent research predominantly employs MLP or gradient-boosting decision tree for EHR data analysis. Although the transformer is arguably the most promising architecture in the AI field,^32^ its application to EHR data remains relatively unexplored. In this context, we conduct a rigorous comparison of FT-Transformer,^33^ a transformer variant, with MLP and XGBoost^34^ using RIAS in this subsection. The performance of each model is the average of 5 random seeds [0,4] with 5-fold cross-validation on the test dataset. We evaluated each model using AUROC, sensitivity, and F1 score, and the comparative results are presented in Table 2.

**Table 2.** Performance comparison.

	In-hospital mortality			6-month mortality			12-month mortality		
	AUROC	Sensitivity	F1	AUROC	Sensitivity	F1	AUROC	Sensitivity	F1
XGBoost	**0.990 **±** **0.01	0.824 ± 0.02	**0.833 **±** **0.02	**0.939 **±** **0.01	0.652 ± 0.05	**0.693 **±** **0.04	**0.925 **±** **0.01	0.551 ± 0.03	**0.632 **±** **0.02
FT-Transformer	0.986 ± 0.01	**0.922 **±** **0.02	0.823 ± 0.03	0.899 ± 0.01	**0.672 **±** **0.03	0.636 ± 0.03	0.905 ± 0.01	**0.630 **±** **0.52	0.574 ± 0.03
MLP	0.982 ± 0.01	0.820 ± 0.04	0.785 ± 0.03	0.900 ± 0.02	0.552 ± 0.05	0.643 ± 0.03	0.812 ± 0.12	0.504 ± 0.01	0.582 ± 0.01

The top results are highlighted in bold.

Table 2 shows that XGBoost achieved the highest scores in the majority of metrics and tasks evaluated, underscoring its robust performance. In contrast, the MLP, despite its widespread use, did not show competitive results in this analysis. Notably, the FT transformer demonstrated superior performance in sensitivity, while also delivering results comparable to XGBoost in the remaining metrics. This highlights the potential benefits of integrating transformer models into AI tasks where high sensitivity is paramount and may offer a compelling alternative to the traditional use of MLP in multi-modal healthcare AI systems.^35^^,^^36^

### Global explanations using RIAS

**Figure 3.**
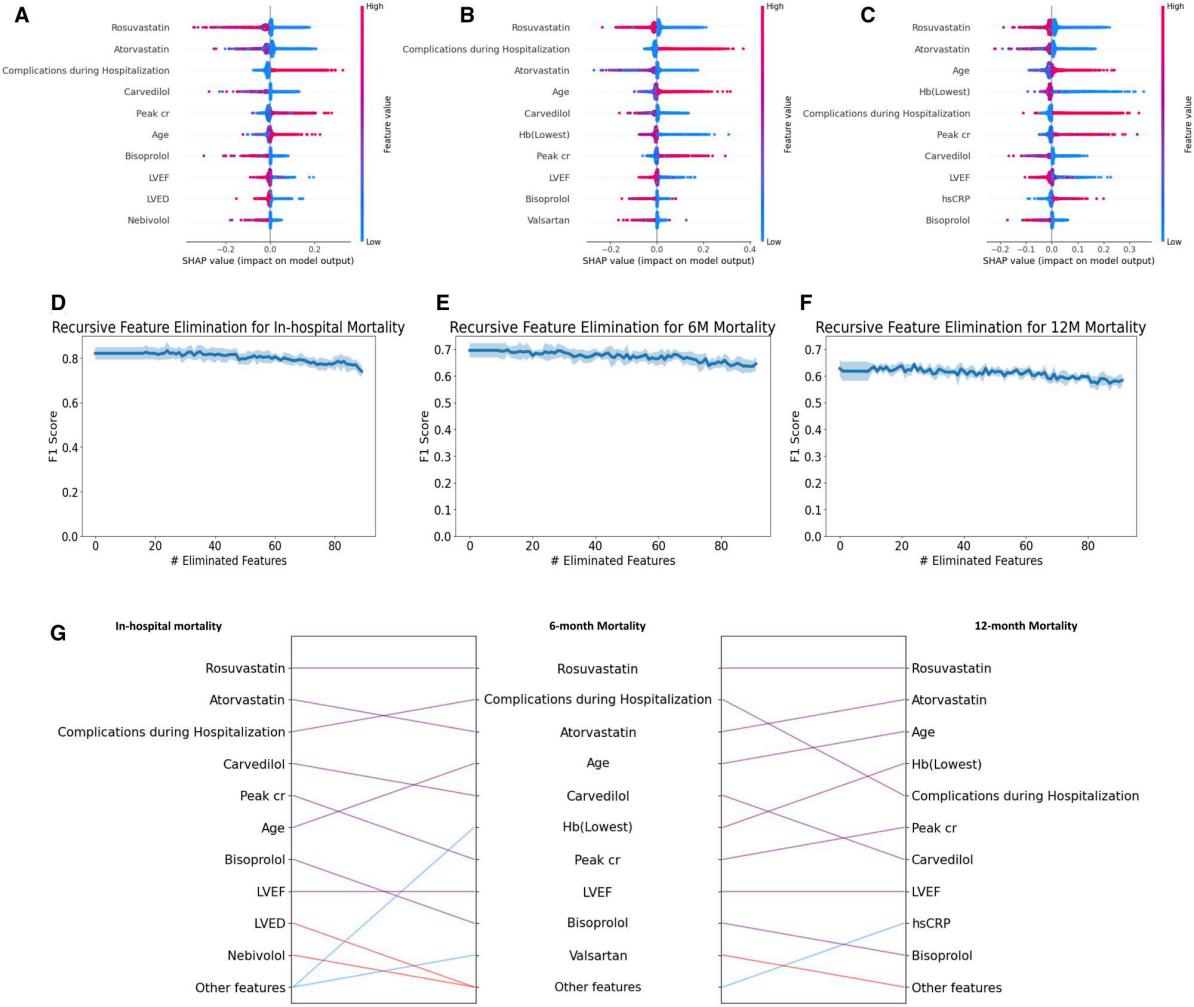
Global Explanations based on SHAP. (A) In-hospital mortality (B) 6-month mortality (C) 12-month mortality. (D–F) represent the F1 scores for recursive feature elimination for in-hospital mortality, 6-month mortality, and 12-month mortality, respectively. The shaded regions indicate the standard deviation over 5 random seeds. (G) Comparison of important features over different periods. The purple line indicates that the feature is included in the top 10 important features of both tasks. The blue and red lines indicate that the feature is included in the top 10 features of one task, but not in the top 10 important features of other tasks.

**Figure 4.**
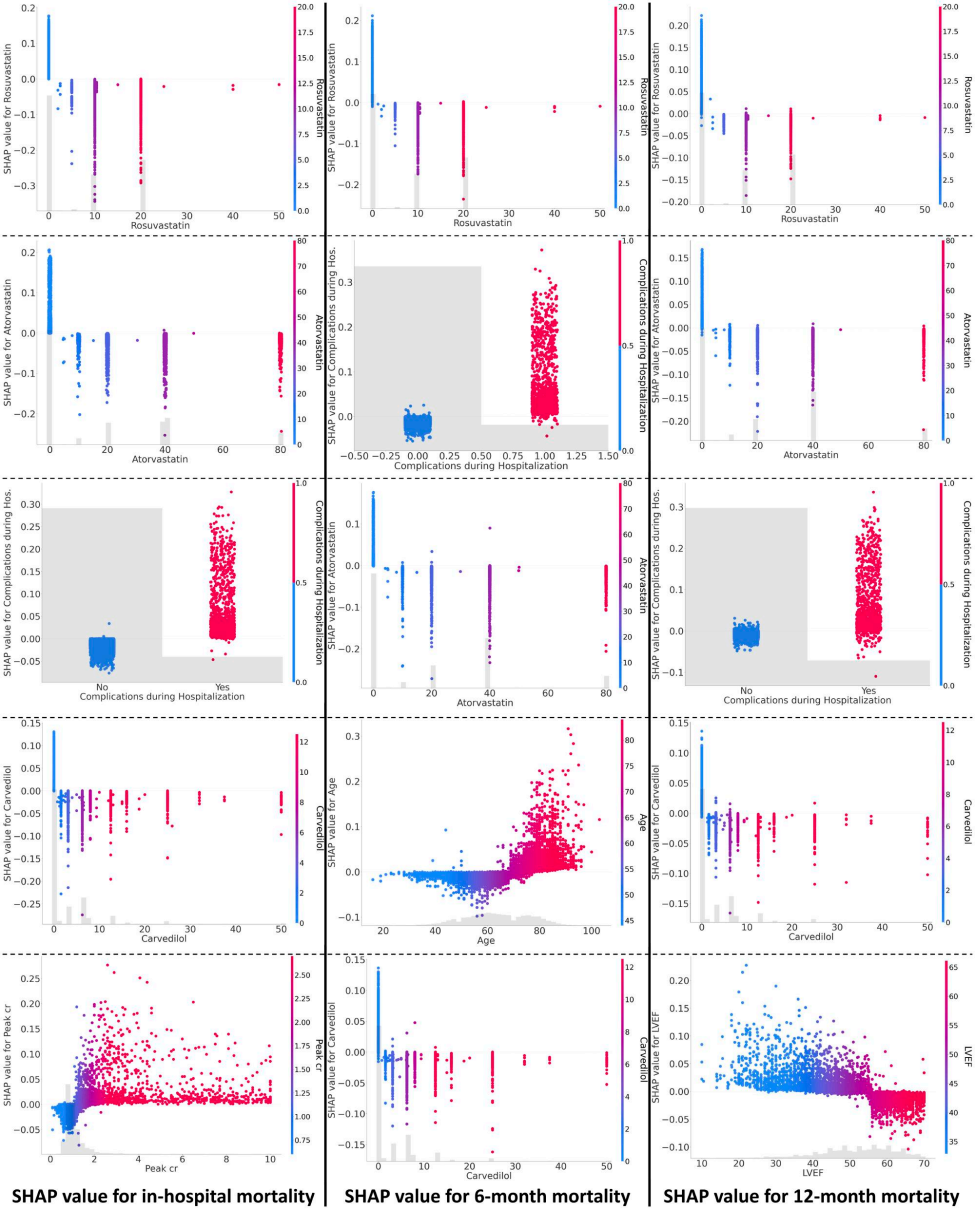
Global explanations of the top 5 most important features for each task. The higher the SHAP value, the greater the contribution to mortality, and each dot represents the individual patient.

We summarize the global contributions of the top 10 most important features for each task in Figure 3A-C and visualize the global explanations of the top 5 most important features for each task in Figure 4. Our findings reveal that statin-based medications and beta-blockers are consistently crucial in mortality predictions over different time periods. Additionally, complications during hospitalization and patient age are identified as significant factors influencing mortality throughout all periods assessed. The global explanations of RIAS were verified using recursive feature elimination (RFE) methods and XGBoost. XGBoost was tuned for 50 trials using Optuna for each feature subset, and the most unimportant feature was recursively discarded. As shown in Figure 3D-F, the consistent performance during RFE proves the global explanations of RIAS. We also present the changes over the time periods in Figure 3G. Rosuvastatin and Atorvastatin, statin-class drugs used to manage high cholesterol levels, appear to be associated with mortality across all time periods, and age seems to have a greater influence on mortality at 6 and 12 months. In contrast, beta-blocker usage and complications during hospitalization held a higher ranking in early post-AMI mortality prediction but diminished to a lower ranking in 12-month mortality, with a reduction in its significance. Cardiac function indicators, such as left ventricular end-diastolic dimension, left ventricular ejection fraction (LVEF), and peak creatinine levels (Peak cr), play a significant role.

### Local explanations and counterfactual examples using RIAS on realistic scenarios

In this subsection, we present the local explanations and counterfactual examples using RIAS within several realistic scenarios. The presentations consist of the following stages. First, RIAS reports its prediction with the likelihood for a given patient along with a local explanation. The likelihood, represented by a central value ranging from 0 to 1, indicates the likelihood of an event occurring, with 0 indicating unlikely and 1 indicating certain. Additionally, the local explanation displays the contribution of each feature to the prediction. Next, a clinician determines the patient’s required recovery plan to achieve the desired outcome. Lastly, RIAS generates counterfactual examples and provides its prediction along with the likelihood for clinicians to assess the effectiveness of the clinical decision, at least regarding its impact on the AI’s outcome. It is important to note that the likelihood generated by our system can be interpreted as the actual likelihood since the RIAS calibrates the confidence of the AI model—which represents the likelihood—to accurately mirror the true correctness likelihood. For the presentations, we select suitable patients for a specific scenario from the test dataset. The details regarding the patients are shown in the supplementary Appendix.

#### Scenario 1: administration of beta-blocker upon discharge

**Figure 5.**
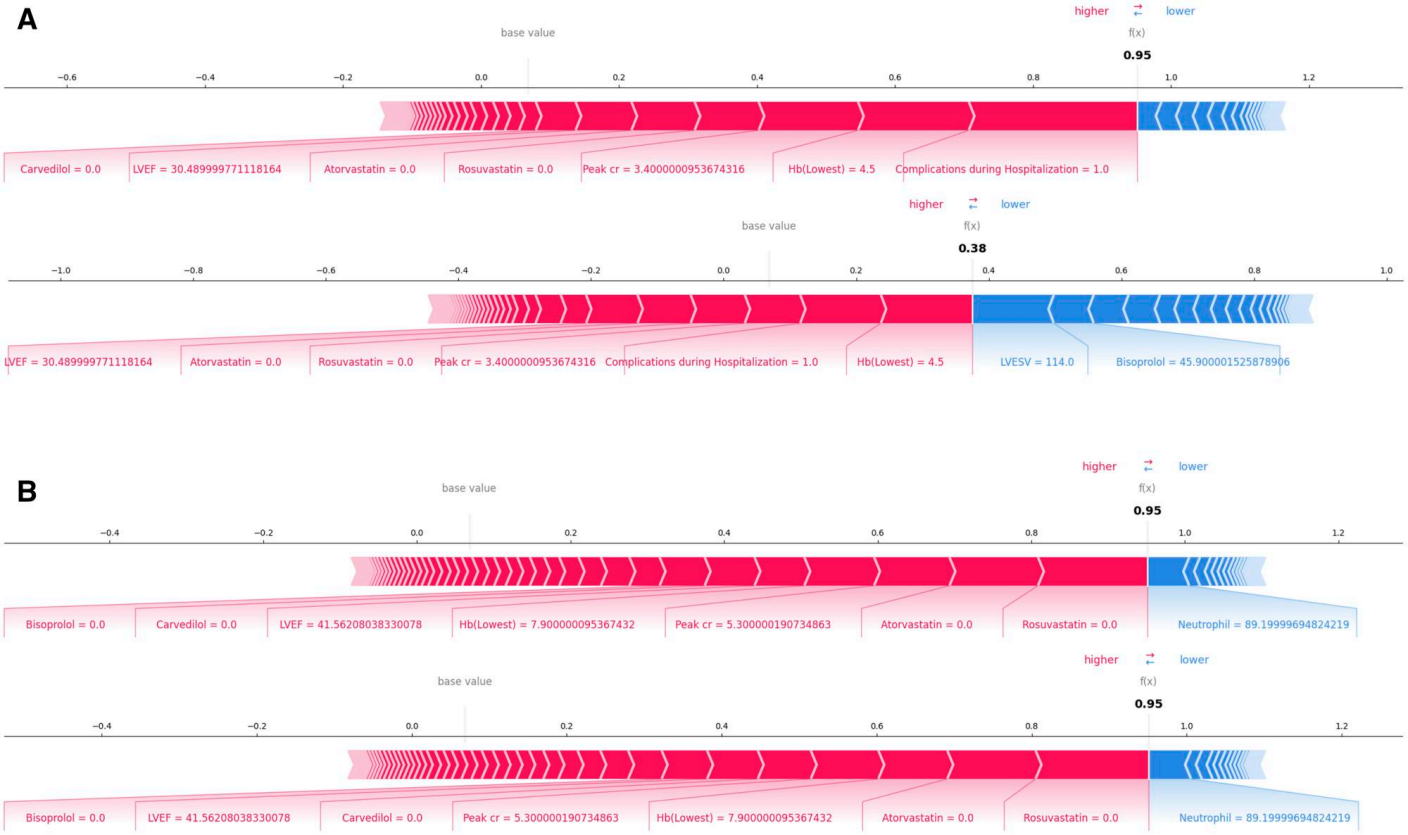
Counterfactual examples with local explanations. (A) The change in mortality without and with beta-blocker prescription when left ventricular ejection fraction is less than 40% (B) The change in mortality according to the absence and presence of clopidogrel, one of the platelet aggregation inhibitors. The bold numbers are the predicted likelihood (f(x)), while the base value is the expectation of the training cohort. Features are represented by arrows that push results to high mortality (right arrows) or low mortality (left arrows). The length of the arrows is proportional to the SHAP values of the relevant features for each prediction. Less important features are omitted for visualization.^41^

Consider a 62-year-old male patient with ST-segment elevation myocardial infarction (STEMI), 3-vessel disease, initially received thrombolysis and then successfully treated with partial revascularization for left anterior descending (LAD) artery, who does not use any beta-blocker or statin as discharge medication. This patient exhibits compromised cardiac function (LVEF 30%) for large anterior infarction, along with unfavorable laboratory findings (peak Cr 3.4 mg/dL, lowest Hb 4.5 g/dL), and experienced complications during hospitalization, which appears to be linked to thrombolysis treatment. Our system predicts a 95% likelihood of mortality for this patient after 12 months. For this patient, mortality was attributed to complications, including significantly reduced hemoglobin and elevated Peak cr. Contributing factors also involved not taking statins, having a low LVEF, and the absence of prescribed beta-blockers in sequence. Then, the clinician verifies the decision using RIAS to produce counterfactual examples of the usage of beta-blockers. As shown in Figure 5A, RIAS finds out the counterfactual example where patients may have a lower mortality likelihood after 12 months. It clarifies that discharging 4.59mg of medication Bisoprolol contributes to decreasing the likelihood of death from 95% to 38%. The use of beta-blockers as part of the discharge medication regimen for patients with AMI has been well-established as an effective treatment for reducing adverse cardiovascular events, with a strong foundation in multiple randomized controlled trials spanning from the pre-reperfusion era to the present. In the contemporary era of reperfusion and potent antiplatelet agents, the use of beta-blockers has become primarily focused on AMI patients with reduced LVEF.^37–39^ As an illustrative example, RIAS accurately anticipated the impact of beta-blockers in reducing mortality. The findings align with current guidelines and prevailing opinions on the matter, and bolster clinical decision-making.

#### Scenario 2: administration of clopidogrel upon discharge

Consider a 69-year-old male with a history of hypertension, presenting with non-ST-segment elevation myocardial infarction (NSTEMI) and 3-vessel disease that was successfully treated with early invasive revascularization only for culprit vessel of left circumflex (LCX) artery, resulting in a final LVEF of 41%. During hospitalization, he experienced acute kidney injury (with the initial serum creatinine level at 1.2 mg/dL escalating to 5.3 mg/dL), a significant drop in hemoglobin indicating a major bleeding event, and atrial fibrillation. Notably, he has not prescribed P2Y12 inhibitors and statins upon discharge, both of which are essential for AMI treatment.^40^ In this scenario, the patient seems to be prescribed only an anticoagulant instead of a dual or mono-antiplatelet agent and anticoagulant for AMI and combined atrial fibrillation due to a significant active bleeding event. He was predicted to die after 12 months with a 95% using our system. For this patient, the most critical factors influencing his mortality prediction were the absence of statin use, highly elevated creatinine, significantly reduced hemoglobin, low LVEF, and the lack of prescribed beta-blockers in sequence. The clinician wants to verify whether the usage of clopidogrel can decrease the likelihood of death or not. Therefore, the clinician uses the RIAS and the system concludes that the usage of clopidogrel does not contribute to the decrease in the death likelihood. According to the system, the likelihood of death still remains at 95% with the usage of clopidogrel (in Figure 5B). The results advise clinicians to prescribe statin/beta-blockers and address the bleeding source, manage kidney injury, or consider clopidogrel use after ad hoc revascularization of the nonculprit vessel to reduce mortality.

We also present an additional realistic scenario with RIAS regarding SGLT2 inhibitors in supplementary Appendix.

## Discussion

In this study, we introduced RIAS—which is a RIAS—for clinicians. Then, we applied RIAS to several tasks predicting mortality following AMI using prospective large multicenter registry data. While recent studies demonstrated that contemporary deep-learning models outperform traditional regression-based prognosis prediction models and earlier ML algorithms, RIAS shows superior results over recent studies. Concretely, Kwon et al. demonstrated that a deep-learning-based model exhibited good performance with an AUROC of 0.870 to 0.905 in predicting long-term mortality after AMI using the same cohort as the current study.^42^ Oliveira et al. utilized the Portuguese AMI registry to assess various ML models for predicting 12-month mortality, resulting in an AUROC of 0.89 and a recall of 0.9.^43^ Notably, our RIAS system exhibited comparable effectiveness when compared with presently prevalent deep-learning models, achieving an AUROC of 0.925 and an F1 score of 0.632 for 12-month mortality. The system gained the most benefit in predicting in-hospital mortality with an AUROC of 0.990 and an F1 score of 0.833. Furthermore, we rigorously investigated employing the transformer-based model, FT-Transformer, over XGBoost and MLP for predicting clinical outcomes in AMI, which is the first investigation with our best knowledge. Our finding reveals the strengths of FT-Transformer, especially its sensitivity, and these results are inlined with contemporary studies using transformer on other various predicting tasks.^44–46^

We also potentially resolved the challenges for clinicians seeking trust and meaningful insights from AI systems due to their “black-boxed operation”—making predictions without providing any explanations, and the discrepancy between predicted likelihood and the true correctness likelihood. Various XAI studies were suggested to address this issue by identifying variables as important features for predicting mortality using SHAP values.^43^^,^^47^^,^^48^ However, each patient represents specific values of variables, so features from a dataset do not quantitatively predict the specific patient’s outcome in real-time. Clinicians may still hesitate if the results are not representative of each individual case, even when provided with explainable data. Furthermore, the problem of predicted mortality differing significantly from the actual likelihood still remains. RIAS has resolved these issues by providing global (overall) and local (individual patient) explanations based on SHAP values and reliable predictions which can be interpreted as the actual likelihood through confidence calibration. In addition, RIAS provides more profound insights into desired outcomes through “what if ∼” counterfactual explanations—scenarios where one or more variables are changed—and demonstrates how these changes would have led to a different decision or prediction from the model. In realistic scenarios, the likelihood of predicted mortality can be altered by modifying variables. Hence, RIAS’s counterfactual explanations empower clinicians to simulate patient scenarios under diverse conditions (eg, changing treatment prescribed at discharge). These functionalities highlight significant strengths in our algorithm, emphasizing its precision in predictions.

The implications of RIAS can be summarized as follows: (1) expediting the development of a new AI system with a different dataset and model; (2) enabling the use of predictions as actual likelihoods; (3) facilitating systematic studies on features contributing to prognosis, both globally and locally, based on SHAP values; (4) offering “what if” scenarios for patient treatment applications.

However, it is important to note several limitations in this study. The algorithm excludes certain crucial indicators for in-hospital mortality and morbidity, such as the symptom-to-hospital visit and door-to-balloon time, which are commonly considered in most AMI studies.^49^ Instead, our study cohort features well-defined variables, encompassing angiographic, laboratory, hemodynamic, and imaging findings. It exhibits consistent characteristics with other contemporary AMI studies, with half of the patients being STEMI patients, 5% experiencing cardiogenic shock, and nearly all patients undergoing PCI and receiving optimal medical therapies thereafter. Our study results demonstrated comparable accuracy to previous studies in short-term mortality using concurrent AMI populations and the most current ML models. However, for 12-month mortality, the model does not include time-varying variables and follow-up characteristics, leading to lower accuracy compared to short-term mortality prediction. Another limitation of our study is the absence of validation on an external cohort for our results. In addition, variables related to medication in this system are based solely on discharge medication information and do not include follow-up medication details. The discontinuation of evidence-based medication is a robust predictor of long-term mortality after AMI. We also were not able to capture other time-variant variables throughout the follow-up period. These factors may compromise the accuracy of the system for 12-month mortality prediction. In addition, RIAS can help improve trust in black-box AI, but the trustworthiness of RIAS depends on the trained classifier. All explanations of RIAS assume that the trained classifier always provides accurate predictions, but there is a risk of incorrect predictions. Furthermore, our system does not fully address other dimensions commonly associated with Trustworthy AI, such as ethical considerations and bias mitigation. These areas, while not directly tackled in our current study, are crucial for the broader discourse on AI’s role in healthcare. Finally, although we present several realistic scenarios using RIAS for clinicians, we have not thoroughly studied the effect of this system on clinicians. Therefore, the actual impact of this system on clinical decision-making in real-world practice needs to be further investigated.

## Conclusion

Predicting mortality after AMI is critical for timely and personalized interventions to reduce AMI mortality and hinder heart failure progression. However, there is a lack of RIASs that clinicians can trust. In this study, we propose the RIAS, an end-to-end framework designed to address these prevailing challenges. With RIAS, we rigorously compare various models on AMI prognosis datasets and present the contributions of each feature to global and local explanations based on SHAP values, in particular revealing the influence of previously known features that are considered important for clinical decision-making. Furthermore, we demonstrate the applicability of “what if” scenarios to validate personalized treatment plans using RIAS in diverse, realistic settings with reliable predictions that can be considered the actual likelihood. Although our study primarily applies RIAS to AMI prognosis datasets using XGBoost, the framework is designed with broader applicability in mind, being model-agnostic and dataset-agnostic. Future work includes exploring broader applications of RIAS for the healthcare domain and practical evaluation of the trustworthiness of RIAS with clinicians.

## Supplementary Material

ocae114_Supplementary_Data

## Data Availability

The KAMIR dataset generated and/or analyzed during the current study cannot be shared publicly due to its inclusion of patient health information protected by the Health Insurance Portability and Accountability Act, but will be shared on reasonable request to the corresponding author.

## References

[1] Tsao CW , Aday AW, Almarzooq ZI, et al; American Heart Association Council on Epidemiology and Prevention Statistics Committee and Stroke Statistics Subcommittee. Heart disease and stroke statistics—2023 update: a report from the American Heart Association. Circulation. 2023;147(8):e93-e621. 36695182 10.1161/CIR.0000000000001123 PMC12135016

[2] Jenča D , Melenovský V, Stehlik J, et al Heart failure after myocardial infarction: incidence and predictors. ESC Heart Fail. 2021;8(1):222-237. 33319509 10.1002/ehf2.13144 PMC7835562

[3] Antman EM , Cohen M, Bernink PJLM, et al The TIMI risk score for unstable angina/non-ST elevation MI: a method for prognostication and therapeutic decision making. JAMA. 2000;284(7):835-842. 10938172 10.1001/jama.284.7.835

[4] Morrow DA , Antman EM, Charlesworth A, et al TIMI risk score for ST-elevation myocardial infarction: a convenient, bedside. clinical score for risk assessment at presentation: an intravenous nPA for treatment of infarcting myocardium early II trial substudy. Circulation. 2000;102(17):2031-2037. 11044416 10.1161/01.cir.102.17.2031

[5] Abu-Assi E , Garćıa-Acũna JM, Peña-Gil C, et al Validation of the GRACE risk score for predicting death within 6 months of follow-up in a contemporary cohort of patients with acute coronary syndrome. Rev Esp Cardiol. 2010;63(6):640-648. 20515621 10.1016/s1885-5857(10)70138-9

[6] Granger CB , Goldberg RJ, Dabbous O, et al; Global Registry of Acute Coronary Events Investigators. Predictors of hospital mortality in the global registry of acute coronary events. Archiv Intern Med. 2003;163(19):2345-2353. 10.1001/archinte.163.19.2345 14581255

[7] Fox KA , FitzGerald G, Puymirat E, et al Should patients with acute coronary disease be stratified for management according to their risk? Derivation, external validation and outcomes using the updated GRACE risk score. BMJ Open. 2014;4(2):e004425. 10.1136/bmjopen-2013-004425 PMC3931985 24561498

[8] Amsterdam EA , Wenger NK, Brindis RG, et al 2014 AHA/ACC guideline for the management of patients with non-ST-elevation acute coronary syndromes: a report of the American College of Cardiology/American Heart Association Task Force on Practice Guidelines. J Am Coll Cardiol. 2014;64(24):e139-e228. 25260718 10.1016/j.jacc.2014.09.017

[9] Lee W , Lee J, Woo SI, et al Machine learning enhances the performance of short and long-term mortality prediction model in non-ST-segment elevation myocardial infarction. Sci Rep. 2021;11(1):12886. 34145358 10.1038/s41598-021-92362-1 PMC8213755

[10] Khera R , Haimovich J, Hurley N, et al Use of machine learning models to predict death after acute myocardial infarction. JAMA Cardiol. 2021;6(6):633-641. 33688915 10.1001/jamacardio.2021.0122 PMC7948114

[11] Gale CP , Stocken DD, Aktaa S, et al Effectiveness of GRACE risk score in patients admitted to hospital with non-ST elevation acute coronary syndrome (UKGRIS): parallel group cluster randomised controlled trial. BMJ. 2023;381:e073843. 37315959 10.1136/bmj-2022-073843 PMC10265221

[12] Samad M , Ulloa A, Wehner G, et al Predicting survival from large echocardiography and electronic health record datasets: optimization with machine learning. JACC Cardiovasc Imaging. 2019;12(4):681-689. 29909114 10.1016/j.jcmg.2018.04.026 PMC6286869

[13] Kim T , Kim M, Lee HW, et al One year mortality prediction in heart failure using feature selection and missing value imputation in deep learning. In: *2023 IEEE International Conference on Big Data and Smart Computing (BigComp).* 2023.

[14] Adadi A , Berrada M. Peeking inside the black-box: a survey on explainable artificial intelligence (XAI). IEEE Access. 2018;6:52138-52160.

[15] Cadario R , Longoni C, Morewedge CK. Understanding, explaining, and utilizing medical artificial intelligence. Nat Hum Behav. 2021;5(12):1636-1642. 34183800 10.1038/s41562-021-01146-0

[16] Quinn TP , Jacobs S, Senadeera M, et al The three ghosts of medical AI: can the black-box present deliver? Artif Intell Med. 2022;124:102158. 34511267 10.1016/j.artmed.2021.102158

[17] Zhang Y , Liao QV, Bellamy RK. Effect of confidence and explanation on accuracy and trust calibration in AI-assisted decision making. In: *Proceedings of the 2020 Conference on Fairness, Accountability, and Transparency*. 2020:295-305.

[18] Asan O , Bayrak AE, Choudhury A. Artificial intelligence and human trust in healthcare: focus on clinicians. J Med Internet Res. 2020;22(6):e15154. 32558657 10.2196/15154 PMC7334754

[19] Cutillo CM , Sharma KR, Foschini L, et al; Machine Intelligence in Healthcare Workshop Working Group. Machine intelligence in healthcare—perspectives on trustworthiness, explainability, usability, and transparency. NPJ Digit Med. 2020;3(1):47. 32258429 10.1038/s41746-020-0254-2 PMC7099019

[20] Naiseh M , Al-Thani D, Jiang N, et al How the different explanation classes impact trust calibration: the case of clinical decision support systems. Int J Hum Comput Stud. 2023;169:102941.

[21] Markus AF , Kors JA, Rijnbeek PR. The role of explainability in creating trustworthy artificial intelligence for health care: a comprehensive survey of the terminology, design choices, and evaluation strategies. J Biomed Inform. 2021;113:103655. 33309898 10.1016/j.jbi.2020.103655

[22] Tonekaboni S , Joshi S, McCradden MD, et al What clinicians want: contextualizing explainable machine learning for clinical end use. In: *Machine Learning for Healthcare Conference*. PMLR; 2019:359-380.

[23] Kim Y , Ahn Y, Cho M, et al Current status of acute myocardial infarction in Korea. Korean J Intern Med. 2018;34(1):1-10. 30612415 10.3904/kjim.2018.381 PMC6325441

[24] Akiba T , Sano S, Yanase T, et al Optuna: a next-generation hyperparameter optimization framework. In: *Proceedings of the 25th ACM SIGKDD International Conference on Knowledge Discovery & Data Mining*. 2019:2623-2631.

[25] Payrovnaziri SN , Chen Z, Rengifo-Moreno P, et al Explainable artificial intelligence models using real-world electronic health record data: a systematic scoping review. J Am Med Inform Assoc. 2020;27(7):1173-1185. 32417928 10.1093/jamia/ocaa053 PMC7647281

[26] Lundberg SM , Lee SI. A unified approach to interpreting model predictions. In: *Advances in Neural Information Processing Systems*. 2017;30.

[27] Das A , Rad P. 2020. Opportunities and challenges in explainable artificial intelligence (XAI): a survey, arXiv, arXiv:200611371, preprint: not peer reviewed.

[28] Guidotti R. Counterfactual explanations and how to find them: literature review and benchmarking. Data Min Knowl Disc. 2022:1-55.

[29] Mothilal RK , Sharma A, Tan C. Explaining machine learning classifiers through diverse counterfactual explanations. In: *Proceedings of the 2020 Conference on Fairness, Accountability, and Transparency*. 2020:607-617.

[30] Karimi AH , Sch¨olkopf B, Valera I. Algorithmic recourse: from counterfactual explanations to interventions. In: *Proceedings of the 2021 ACM Conference on Fairness, Accountability, and Transparency*. 2021:353-362.

[31] Guo C , Pleiss G, Sun Y, et al On calibration of modern neural networks. In: *International Conference on Machine Learning*. PMLR; 2017:1321-1330.

[32] Vaswani A , Shazeer N, Parmar N, et al Attention is all you need. Adv Neural Inform Process Syst. 2017;30:5998-6008.

[33] Gorishniy Y , Rubachev I, Khrulkov V, et al, et al Revisiting deep learning models for tabular data. In: Ranzato M, Beygelzimer A, Dauphin Y, eds. Advances in Neural Information Processing Systems. Vol 34. Curran Associates, Inc.; 2021:18932-18943.

[34] Chen T , Guestrin C. XGBoost: a scalable tree boosting system. In: *Proceedings of the 22nd ACM SIGKDD International Conference on Knowledge Discovery and Data Mining KDD ’16*. Association for Computing Machinery; 2016:785-794.

[35] Mohsen F , Ali H, El Hajj N, et al Artificial intelligence-based methods for fusion of electronic health records and imaging data. Sci Rep. 2022;12(1):17981. 36289266 10.1038/s41598-022-22514-4 PMC9605975

[36] Zhi Z , Elbadawi M, Daneshmend A, et al Multimodal diagnosis for pulmonary embolism from EHR data and CT images. In: *2022 44th Annual International Conference of the IEEE Engineering in Medicine & Biology Society (EMBC)*. IEEE; 2022:2053-2057. 10.1109/EMBC48229.2022.9871041 36086373

[37] Investigators C , et al Effect of carvedilol on outcome after myocardial infarction in patients with left-ventricular dysfunction: the CAPRICORN randomised trial. Lancet. 2001;357(9266):1385-1390. 11356434 10.1016/s0140-6736(00)04560-8

[38] Byrne RA , Rossello X, Coughlan J, et al; ESC Scientific Document Group. 2023 ESC guidelines for the management of acute coronary syndromes: developed by the task force on the management of acute coronary syndromes of the European Society of Cardiology (ESC). Eur Heart J. 2023;44(38):3720-3826. 37622654 10.1093/eurheartj/ehad191

[39] Watanabe H , Ozasa N, Morimoto T, et al; CAPITAL-RCT Investigators. Long-term use of carvedilol in patients with ST-segment elevation myocardial infarction treated with primary percutaneous coronary intervention. PLoS One. 2018;13(8):e0199347. 30153268 10.1371/journal.pone.0199347 PMC6112626

[40] Members WC , Lawton JS, Tamis-Holland JE, et al 2021 ACC/AHA/SCAI guideline for coronary artery revascularization: a report of the American College of Cardiology/American Heart Association Joint Committee on Clinical Practice Guidelines. J Am Coll Cardiol. 2022;79(2):e21-e129. 34895950 10.1016/j.jacc.2021.09.006

[41] Chieregato M , Frangiamore F, Morassi M, et al A hybrid machine learning/deep learning COVID-19 severity predictive model from CT images and clinical data. Sci Rep. 2022;12(1):4329. 35288579 10.1038/s41598-022-07890-1 PMC8919158

[42] Kwon J , Jeon KH, Kim HM, et al Deep-learning-based risk stratification for mortality of patients with acute myocardial infarction. PLoS One. 2019;14(10):e0224502. 31671144 10.1371/journal.pone.0224502 PMC6822714

[43] Oliveira M , Seringa J, Pinto FJ, et al Machine learning prediction of mortality in acute myocardial infarction. BMC Med Inform Decis Mak. 2023;23(1):70-16. 37072766 10.1186/s12911-023-02168-6 PMC10111317

[44] Huang Y , Wang M, Zheng Z, et al Representation of time-varying and time-invariant EMR data and its application in modeling outcome prediction for heart failure patients. J Biomed Inform. 2023;143:104427. 37339714 10.1016/j.jbi.2023.104427

[45] Ansari Y , Mourad O, Qaraqe K, et al Deep learning for ECG arrhythmia detection and classification: an overview of progress for period 2017-2023. Front Physiol. 2023;14:1246746. 37791347 10.3389/fphys.2023.1246746 PMC10542398

[46] Allou N , Allyn J, Provenchere S, et al; EpiCard Investigators. Clinical utility of a deep-learning mortality prediction model for cardiac surgery decision making. J Thorac Cardiovasc Surg. 2023;166(6):e567-e578. 36858843 10.1016/j.jtcvs.2023.01.022

[47] Moore A , Bell M. XGBoost, a novel explainable AI technique, in the prediction of myocardial infarction: a UK Biobank Cohort Study. Clin Med Insights Cardiol. 2022;16:11795468221133611. 36386405 10.1177/11795468221133611 PMC9647306

[48] Tarabanis C , Kalampokis E, Khalil M, et al Explainable SHAP-XGBoost models for in-hospital mortality after myocardial infarction. Cardiovasc Digit Health J. 2023;4(4):126-132. 37600443 10.1016/j.cvdhj.2023.06.001 PMC10435947

[49] Reed GW , Rossi JE, Cannon CP. Acute myocardial infarction. Lancet. 2017;389(10065):197-210. 27502078 10.1016/S0140-6736(16)30677-8

