# Supplementary material for: Acute myocardial infarction prognosis prediction with reliable and interpretable artificial intelligence system
Source: J Am Med Inform Assoc. 2024 May 28;31(7):1540–50. doi: 10.1093/jamia/ocae114 (PMC11187491; doi:10.1093/jamia/ocae114)
Supplement: ocae114_Supplementary_Data [file ocae114_supplementary_data.pdf]

A. FEATURE ENGINEERING AND DATA PROCESSING

We excluded certain samples due to loss of follow-up, resulting in a dataset of 15,629 samples used to predict in-hospital mortality, 14,613 samples for 6-month mortality, and 14,260 samples for 12-month mortality. The KAMIR dataset has originally 614 features, but most of them are considered irrelevant to or hindered the mortality prediction tasks by clinical experts. Therefore, we excluded the unnecessary features, leaving 114 features. We identified several outliers caused by potential data collection issues and converted them to missing values for imputation later. Then, we excluded features with a missing rate of 40% or more, leaving 97 features. We impute the remaining missing values using a powerful statistical technique called MICE [1]. The data processing flowchart is shown in Fig. A1.

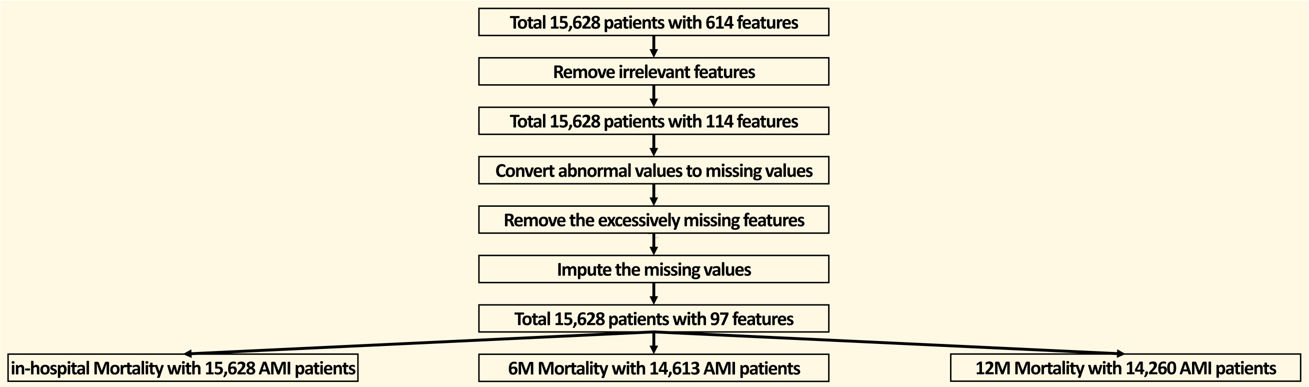

Fig. A1: Feature Engineering and Data Processing

B. GRACE SCORE FOR 12-MONTH MORTALITY

Table A1. A summary of the risk level using the GRACE score

Mortality / Risk Level	Low	Intermediate	High
Live	200 (1.5%)	12714 (95.2%)	440 (3.3%)
Death	37 (4.1%)	840 (92.7%)	29 (3.2%)

C. EXPECTED CALIBRATION ERROR (ECE)

The Expected Calibration Error (ECE) [2] quantifies the discrepancy between a model's predicted confidence (i.e. likelihood) and its true accuracy. To compute the ECE, predictions are grouped into M bins of equal sizes based on their confidence, and the difference between the average accuracy and average confidence for each bin is determined. Formally, the ECE is given by:

$$ECE = \sum_{m=1}^{M} \frac{|B_m|}{n} \left| acc(B_m) - conf(B_m) \right|, \tag{1}$$

where B_m is the set of indices of samples whose prediction confidence falls within interval $I_m = (\frac{m-1}{M}, \frac{m}{M}]$, $|B_m|$ represents the number of predictions in the m_{th} bin, n denotes the total number of samples, and acc and $conf$ denote the average accuracy and average confidence of each bin, respectively.

D. THE SEARCH RANGE OF HYPERPARAMETERS FOR EACH MODEL

Table A2. Hyperparameter search space for XGBoost

Hyperparameter	Search Method	Search Space
max_leaves	suggest_int	[300,4000]
n_estimators	suggest_int	[10,3000]
learning_rate	suggest_uniform	[0,1]
max_depth	suggest_int	[3, 20]
scale_pos_weight	suggest_int	[1, 100]

Table A3. Hyperparameter search space for FT-Transformer

Hyperparameter	Search Method	Search Space
input_embed_dim	suggest_categorical	[16,24,32,48]
embedding_dropout	suggest_uniform	[0.05,0.3]
share_embedding	suggest_categorical	[True, False]
num_heads	suggest_categorical	[1,2,4,8]
num_attn_blocks	suggest_int	[2,10]
transformer_activation	suggest_categorical	[GEGLU, ReGLU, SwiGLU]
use_batch_norm	suggest_categorical	[True, False]
batch_norm_continuous_input	suggest_categorical	[True, False]
learning_rate	suggest_uniform	[0.0001, 0.05]
scheduler_gamma	suggest_uniform	[0.1, 0.95]
scheduler_step_size	suggest_int	[10, 100]

Table A4. Hyperparameter search range for MLP

Hyperparameter	Search Method	Search Space
embedding_dropout	suggest_uniform	[0, 0.2]
layers	suggest_categorical	[128-64-32, 256-128-64, 128-64-32-16, 256-128-64-32]
activation	suggest_categorical	[ReLU, LeakyReLU]
learning_rate	suggest_uniform	[0.0001, 0.05]
scheduler_gamma	suggest_uniform	[0.1, 0.95]
scheduler_step_size	suggest_int	[10, 100]

E. THE IMPACT OF CONFIDENCE CALIBRATION

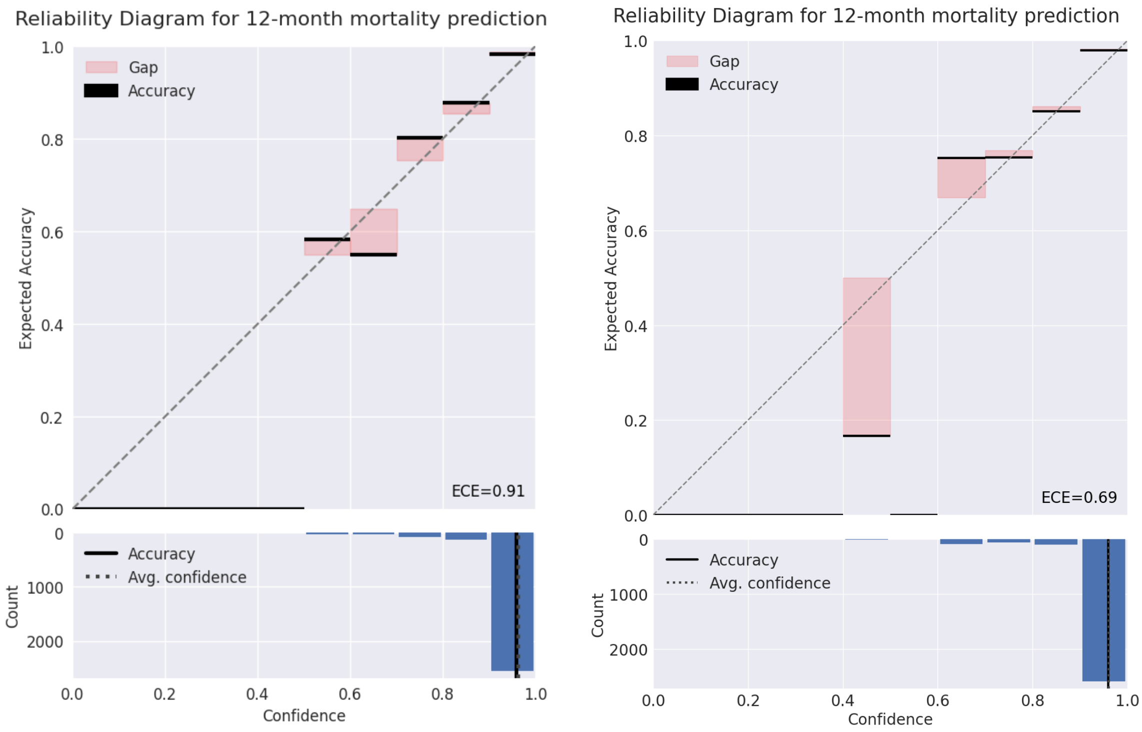

Fig. A2: **(Left)** Before Calibration. **(Right)** After Calibration.

F. ADDITIONAL REALISTIC SCENARIO WITH RIAS

Scenario 3: Discharging Sodium-Glucose Transport Protein 2 (SGLT2) Inhibitors or Not

Consider a 75-year-old female patient with a history of hypertension and diabetes presenting with NSTEMI and a favorable Killip class. She had 3-vessel disease and was suboptimally treated with only medical treatment for the culprit vessel of LCX, resulting in a final good LVEF of 55%. Initially, she had an elevated creatinine of 3.0mg/dL, peaking at 3.3mg/dL, indicating pre-existing diabetic or hypertensive chronic kidney disease. She was not prescribed a statin, beta-blockers, or any ACEi/ARBs as discharge medication. She was predicted to have a 71% chance of mortality after 12 months using our system, primarily due to complications during hospitalization, elevated peak creatinine level, not using statins, and low body weight. The clinician wants to verify whether the usage of SGLT2 inhibitor can make the patient survive or not. Therefore, the clinician uses the RIAS and the system concludes that the usage of 14.859mg of dapagliflozin, one of SGLT2 inhibitors, contributes to the decrease in the likelihood of death. As shown in Figure A3, administering dapagliflozin decreases the likelihood from 71% to 38%. Dapagliflozin was not included as a variable correlated with any mortality event after AMI in this algorithm. The cardioprotective role of SGLT2 inhibitors in diabetes are well known in high risk of atherosclerotic cardiovascular disease including old myocardial infarction [3, 4]. Furthermore, SGLT2 inhibitors have emerged as the gold standard treatment for heart failure, extending beyond patients with reduced ejection fraction, now encompassing those with preserved ejection fraction exceeding 40%, regardless of diabetes [5–8]. Regarding the acute phase of myocardial infarction, for individuals at high risk of

developing heart failure, several ongoing randomized trials are expected to offer conclusive evidence on this matter. The RIAS system has predicted that even AMI patients with diabetes and preserved LVEF would benefit from SGLT2 inhibitors during the acute phase of AMI in terms of reducing mortality. This generates hypotheses and suggestions from the algorithm. Similarly, an analysis of claims data using propensity matching showed that the use of SGLT2 inhibitors during the acute phase of AMI significantly reduced all-cause mortality and hospitalizations for heart failure (the predefined primary endpoint) during a 2.1-year follow-up period [9]. In the findings of this study, the group receiving SGLT2 inhibitors achieved a 34% reduction in the primary endpoint and a 45% decrease in all-cause mortality. In this counterfactual analysis, the system predicted a 33% reduction in likelihood of mortality when using dapagliflozin for one year. The RIAS system provides valuable insights into unresolved issues, such as this scenario, with outcomes closely aligned with real-world data.

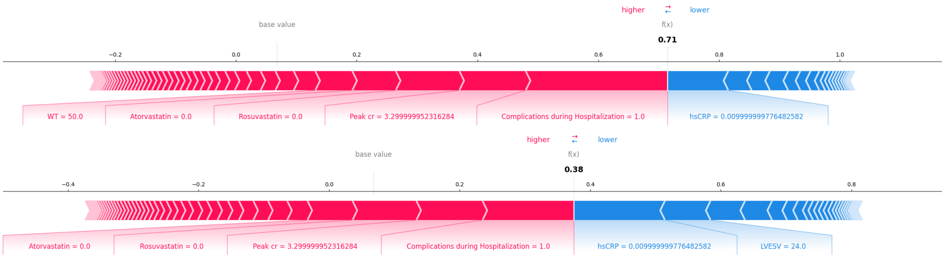

Fig. A3: The change in mortality without and with SGLT2 inhibitor prescription when LVEF is above 40%.

G. DETAILS OF PATIENTS IN REALISTIC SCENARIOS USING RIAS

Feature	Patient 1	Patient 2	Patient 3	Feature	Patient 1	Patient 2	Patient 3
Age	62	69	75	Clopidogrel	1	0 / 1	0
Gender	male	male	female	Prasugurel	0	0	0
HT (cm)	172	160	145	Cilostazol	0	0	0
WT (kg)	68	67	20	Ticagrelor	0	0	0
Smoking History	smoker	never	ex-smoker	Ca-channel blocker	0	0	0
Previous angina before MI index	True	False	False	Fibrate	0	0	0
HTN	False	True	True	Ezetimide	0	0	0
DM	False	False	True	Oral anticoagulant	0	0	0
DL	False	False	False	Bisoprolol	0 / 45.9	0	0
MI	False	False	False	Carvedilol	0	109.4	0
Angina	False	False	False	Nebivolol	0	0	0
HF	False	False	False	Atenolol	0	0	0
CVA	False	False	False	Betaxolol	0	0	0
LVED	True	0	True	Metoprolol	0	0	0
LVEF	30.49	41.56	55.44	Celiprolol	0	0	0
LVESD	32	42	16.3	Perindopril	0	0	0
LVEDD	48	51.44	29.4	Captopril	0	0	0
LVESV	114	64.14	24	Enalapril	0	0	0
LVEDV	164	101.1	55	Ramilpril	0	0	0
SBP	139	0	159	Cilazapril	0	0	0
DBP	78	0	74	Imidapril	0	0	0
HR	81	98	85	Zofenopril	0	0	0
HbA1c	5.5	5.25	5.7	Irbesartan	0	0	0
hsCRP	0.3	3.34	0.01	Candesartan	0	0	0
LDL-c	99	91.62	66	Losartan	0	0	0
HDL-c	54	36.6	21.2	Valsartan	0	0	0
TG	30	83.05	47	Telmisartan	0	0	0
Total cholesterol	168	149.27	170	Olmesartan	0	0	0
WBC	10.4	7.1	8.4	Fimasartan	0	0	0
Neutrophil	85.1	89.2	75.2	Axilsartan	0	0	0
lymphocyte	10.8	7.6	15.7	Eprosartan	0	0	0
Peak Troponin I	395	5.127	1.07	Atorvastatin	0	0	0
Peak CK-MB	574	35.34	24.6	Rosuvastatin	0	0	0
Peak CK	3476	1907	70.9	Simvastatin	0	0	0
Peak cr	3.4	5.3	3.3	Pravastatin	0	0	0
On admission cr	0.7	1.2	3	Fluvastatin(XL)	0	0	0
Glucose (on admission)	98	221.7	262	Fluvastatin	0	0	0
Platelet (on admission)	229	204	249	Pitavastatin	0	0	0
Hb (Lowest)	4.5	7.9	7.8	Empagliflozin	0	0	0
Hb (on admission)	13.7	15.3	10	Dapagliflozin	0	0	0 / 1485.9
STEMI	Primary	Facilitated	Facilitated	omega3	0	0	0
NSTEMI	Early invasive	Early conservative	No	Before Adm	False	False	False
Number of involved vessels	3	3	4	Killip Class at admission	0	1	0
Puncture route	Both	Both	Both	MR grade	1	0	0
Target vessel	No	LAD	LAD	Final diagnosis	STEMI	STEMI	NSTEMI
Pre TIMI flow of target vessel	3	1	1	Complications during Hospital.	True	False	True
Post TIMI flow of target vessel	3	3	2	Lab findings	True	True	True
Treatment of target Vessel	No	No	balloon	IIb/IIIa inhibitors	False	True	False
Result of PCI	Yes	Yes	No	Use of distal protection device	True	False	False
Status of revascularization	Partial	Partial	No	Use of IVUS	True	False	False
Index procedure	None	None	None	Use of FFR	False	False	False
Staged PCI	single PCI	single PCI	single PCI	Use of OCT	False	False	False
PCI at this hospital	True	True	False	PCI in Target Vessel	True	True	False

Table A5. Characteristics of the patients in counterfactual examples. MI=myocardial infarction; STEMI=ST segment elevation MI; NSTEMI=non-ST segment elevation MI; CK=Creatine Kinase; MB=Myocardial Band; hsCRP=high-sensitivity C-Reactive Protein; LDL=Low-Density Lipoprotein; HDL=High-Density Lipoprotein, PCI=percutaneous coronary intervention; IVUS=Intravascular Ultrasound; FFR=Fractional Flow Reserve; OCT=Optical Coherence Tomography

REFERENCES

1. Van Buuren S, and Groothuis-Oudshoorn K. mice: Multivariate Imputation by Chained Equations in R. *Journal of Statistical Software* 2011;45(3):1–67.
2. Naeini MP, Cooper G, and Hauskrecht M. Obtaining well calibrated probabilities using bayesian binning. In: Proceedings of the AAAI conference on artificial intelligence. vol. 29; 2015. .
3. Wiviott SD, Raz I, Bonaca MP et al. Dapagliflozin and cardiovascular outcomes in type 2 diabetes. *New England Journal of Medicine* 2019;380(4):347-57.
4. Zinman B, Wanner C, Lachin JM et al. Empagliflozin, cardiovascular outcomes, and mortality in type 2 diabetes. *New England Journal of Medicine* 2015;373(22):2117-28.

5. McMurray JJ, Solomon SD, Inzucchi SE et al. Dapagliflozin in patients with heart failure and reduced ejection fraction. *New England Journal of Medicine* 2019;381(21):1995-2008.

6. Packer M, Anker SD, Butler J et al. Cardiovascular and renal outcomes with empagliflozin in heart failure. *New England Journal of Medicine* 2020;383(15):1413-24.

7. Anker SD, Butler J, Filippatos G et al. Empagliflozin in heart failure with a preserved ejection fraction. *New England Journal of Medicine* 2021;385(16):1451-61.

8. Solomon SD, McMurray JJ, Claggett B et al. Dapagliflozin in heart failure with mildly reduced or preserved ejection fraction. *New England Journal of Medicine* 2022;387(12):1089-98.

9. Kwon O, Myong JP, Lee Y et al. Sodium-Glucose Cotransporter-2 Inhibitors After Acute Myocardial Infarction in Patients With Type 2 Diabetes: A Population-Based Investigation. *Journal of the American Heart Association* 2023;12(14):e027824.
